# Supplementary figures and images for: Effects of semaglutide on gut microbiota, cognitive function and inflammation in obese mice
Source: PeerJ. 2024 Aug 12;12:e17891. doi: 10.7717/peerj.17891 (PMC11326427; doi:10.7717/peerj.17891)

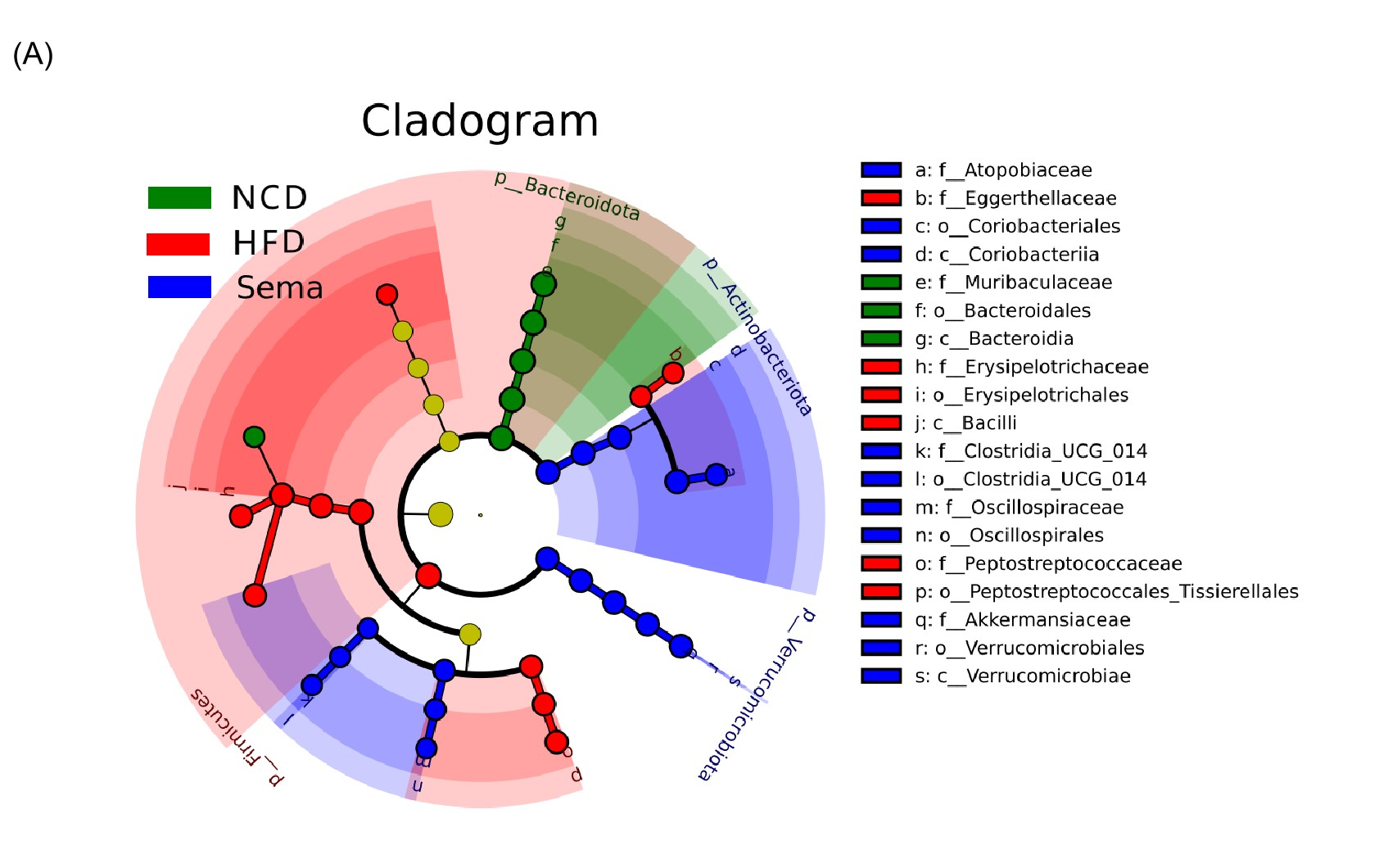

Supplement: Supplemental Information 12 — Different colors indicate different groups. Nodes of different colors indicate the microbes that play an important role in the group represented by the color. From the inside to the outside, each circle is the species at the level of phylum, class, order, family, and genus. [file peerj-12-17891-s012.png]

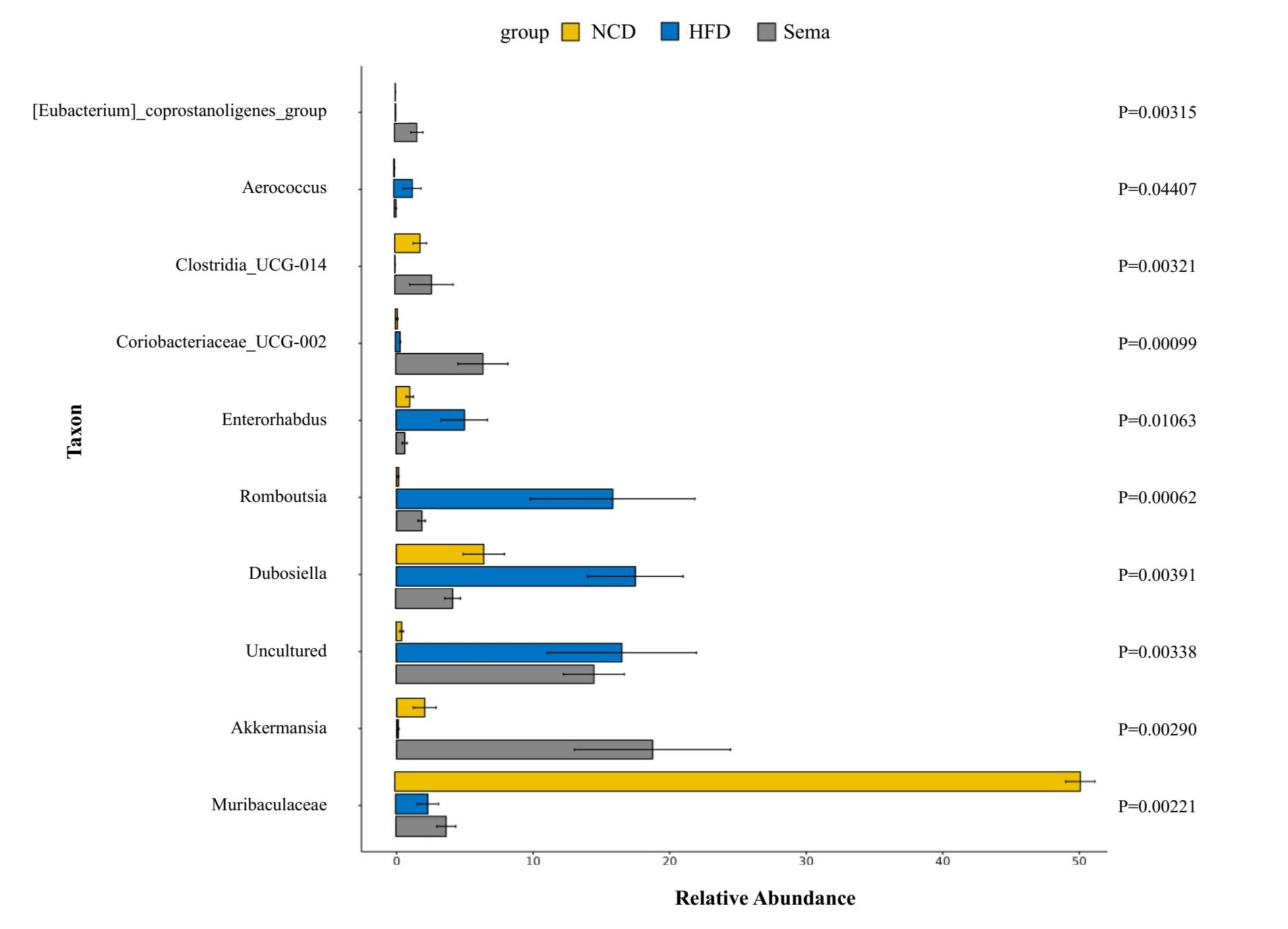

Supplement: Supplemental Information 13 [file peerj-12-17891-s013.png]
